# Melatonin and (−)-Epigallocatechin-3-Gallate: Partners in Fighting Cancer

**DOI:** 10.3390/cells8070745

**Published:** 2019-07-19

**Authors:** Lingyun Zhang, Yufeng He, Ximing Wu, Guangshan Zhao, Ke Zhang, Chung S. Yang, Russel J. Reiter, Jinsong Zhang

**Affiliations:** 1 Laboratory of Redox Biology, State Key Laboratory of Tea Plant Biology and Resources Utilization, School of Tea & Food Science, Anhui Agricultural University, Hefei 230000, China; 2 Department of Chemical Biology, Ernest Mario School of Pharmacy, Rutgers, The State University of New Jersey, Piscataway, NJ 08854, USA; 3 Department of Cellular and Structural Biology, UT Health Science Center, San Antonio, TX 78229, USA

**Keywords:** EGCG, melatonin, cancer, p21, quinoprotein, thioredoxin system

## Abstract

We have demonstrated previously that melatonin attenuates hepatotoxicity triggered by high doses of (−)-epigallocatechin-3-gallate (EGCG) in mice. The current work investigated the influence of melatonin on the oncostatic activity of EGCG in two cancer cell lines, wherein melatonin induced an opposite response of p21. In human tongue cancer TCA8113 cells, melatonin-induced p21 and EGCG-mediated formation of quinoproteins were positively associated with the oncostatic effects of melatonin and EGCG. Melatonin-stimulated an increase in p21 which was correlated with a pronounced nuclear translocation of thioredoxin 1 and thioredoxin reductase 1, both of which are known to induce p21 via promoting p53 trans-activation. Melatonin did not influence the EGCG-mediated increase of quinoprotein formation nor did EGCG impair melatonin-induced p21 up-regulation. Co-treatment with both agents enhanced the cell-killing effect as well as the inhibitory activities against cell migration and colony formation. It is known that p21 also plays a powerful anti-apoptotic role in some cancer cells and confers these cells with a survival advantage, making it a target for therapeutic suppression. In human hepatocellular carcinoma HepG2 cells, melatonin suppressed p21 along with the induction of pro-survival proteins, PI3K and COX-2. However, EGCG prevented against melatonin-induced PI3K and COX-2, and melatonin probably sensitized HepG2 cells to EGCG cytotoxicity via down-regulating p21, Moreover, COX-2 and HO-1 were significantly reduced only by the co-treatment, and melatonin aided EGCG to achieve an increased inhibition on Bcl2 and NFκB. These events occurring in the co-treatment collectively resulted in an enhanced cytotoxicity. In addition, the co-treatment also enhanced the inhibitory activities against cell migration and colony formation. Overall, the results gathered from these two cancer cell lines with a divergent p21 response to melatonin show that the various oncostatic activities of melatonin and EGCG together are more robust than each agent alone, suggesting that they may be useful partners in fighting cancer.

## 1. Introduction

Melatonin is a potent antioxidant with an unequivocal oncostatic property [1,2,3,4,5,6]. Numerous studies have shown that melatonin acts as an antioxidant in normal cells or tissues under oxidative stress, via direct scavenging reactive oxygen species (ROS), inducing antioxidant enzymes or activating nuclear factor erythroid 2-related factor 2 (Nrf2)-dependent antioxidant defense system [3,7,8,9]. However, in cancer cells with higher levels of ROS and concomitant expanded antioxidant arsenal such as aberrantly accumulated Nrf2 and overexpression of DJ1, thioredoxin (Trx) and thioredoxin reductase (TrxR) [10,11,12,13], melatonin no longer plays antioxidant roles but in turn becomes a prooxidant agent to heighten oxidant stress to cause cell death [4,5]. Unlike many recognized antioxidant agents with prooxidant properties, such as selenium, with demonstrated adverse effects at doses required for achieving solid anti-cancer effects [14], melatonin is an exceptional antioxidant with prooxidant activity without documented serious adverse effects [15]. The cell-context dependent feature of melatonin and its uncommonly high safety profile make melatonin a unique molecule for cancer prevention, or cancer adjunctive therapy for attenuating toxic reactions caused by chemotherapy or radiotherapy and sensitizing cancer cells to the therapy [5,16,17,18,19,20,21,22,23,24,25].

Green tea, made from the plant *Camellia sinensis* L., has been consumed in China for over 4000 years and is currently one of the most popular beverages worldwide [26]. In the last three decades, an increasing body of evidence suggests that green tea catechins have health promotion effects, such as alleviation of metabolic syndrome and prevention of neurodegenerative diseases and cancer [27]. Since (−)-epigallocatechin-3-gallate (EGCG) accounts for over half of the catechins in green tea and is the most redox-active tea catechin due to its two ortho-dihydroxy structure, this naturally occurring compound has been used commercially as a dietary supplement. In at least 13 animal models for human carcinogenesis of the lung, oral cavity, esophagus, stomach, small intestine, colorectal, colon, skin, liver, pancreas, bladder, prostate, or mammary glands, EGCG has shown cancer preventive activities [27,28]. Like melatonin, EGCG is also an antioxidant via its direct quenching of ROS or indirect induction of basal and/or Nrf2-dependent antioxidant defense systems [27,29]. On the other hand, at high doses and in certain environments, EGCG can act as a prooxidant owing to its auto-oxidation, resulting in the formation of the superoxide anion and hydrogen peroxide [30]. Unlike melatonin, EGCG at the dose levels that exhibit a solid anti-cancer, anti-obesity or anti-inflammation effects may evoke toxic reactions in certain normal tissues, particular in the liver [31,32,33,34,35,36,37,38]. Thus, a tolerable upper intake level of 300 mg EGCG/person/day for food supplements was issued by France in 2014 and Italy in 2016 [39] and was proposed by some authors in 2017 [40]. Moreover, researchers in Herbalife Nutrition recently suggested a safe intake level of 338 mg EGCG/day for adults [41]. If these doses are commonly accepted and become regulatory dose levels, the cancer preventive potential of EGCG would be largely compromised because many human studies have shown that cancer risk decreases with increasing consumption of green tea [42,43,44,45,46,47,48,49]. Thus, new approaches to mitigate EGCG hepatoxicity and concomitantly increase cancer-inhibitory effects of EGCG are needed. In this regard, we have demonstrated that melatonin can effectively reduce EGCG hepatotoxity in mice. Specifically, melatonin increased survival time of mice treated with a lethal dose of EGCG, attenuated acute liver damage and prevented the down-regulation of hepatic Nrf2 caused by a single administration of a nonlethal but highly toxic dose of EGCG, and mitigated subacute liver injury and hepatic Nrf2 activation induced by multiple administrations of a lower toxic dose of EGCG [50].

Melatonin increases the therapeutic efficacy of many chemotherapeutic drugs by decreasing toxicities and increasing sensitivity of tumors to these therapeutic agents [5,16,17,18,19,20,21,22,23,24,25]. However, whether melatonin would increase the cancer-inhibitory effect of EGCG has not been previously investigated. The goal of the present study was to investigate the influence of melatonin on oncostatic activity of EGCG. In two cancer cell lines examined with diverged p21 response to melatonin, we consistently found that melatonin and EGCG together were more effective than each agent alone in suppressing cell growth, cell migration and colony formation. In combination with the protective effect of melatonin against EGCG hepatotoxicity [50], the present study suggests that the combination of melatonin and EGCG could be a promising means for cancer prevention or therapy.

## 2. Materials and Methods

### 2.1. Reagents

EGCG (>99% purity) was obtained from Yibeijia Tea Technology (Hangzhou, China). Melatonin (>98% purity), nitroblue tetrazolium (NBT) and protease inhibitor cocktail were purchased from Sigma (St. Louis, MO, USA). The primary antibody against β-actin was purchased from Sigma (St. Louis, MO, USA). The primary antibody against γ-H2AX was purchased from Santa Cruz (Dallas, TX, USA). The primary antibody against TrxR1 was offered by Professor Gary F. Merrill (Oregon State University). The other primary antibodies as well as the anti-mouse and anti-rabbit secondary antibodies were purchased from Cell Signaling Technology (Boston, MA, USA). L-glutamine was purchased from Gibco (Grand Island, NY, USA). Fetal bovine serum, RPMI 1640 medium, trypsin, penicillin-streptomycin solutions were purchased from HyClone (Logan, UT, USA). 3-(4,5-Dimethyl-2-thiazolyl)-2,5-diphenyltetrazolium bromide (MTT) was a product of Amresco LLC (Solon, OH, USA). Other chemicals were of the highest grade available.

### 2.2. Cell Lines and Cell Culture

Human tongue squamous cell line TCA8113 was provided by the Key Laboratory of Oral Biomedicine of Shanghai Jiaotong University (Shanghai, China). Human hepatocellular carcinoma (HCC) cell line HepG2 was obtained from the Stem Cell Bank of Chinese Academy of Sciences (Shanghai, China). Both cell lines were cultured in RPMI 1640 medium containing 10% (*v*/*v*) fetal bovine serum, 100 U/mL penicillin and 100 μg/mL streptomycin. The medium for HepG2 cells additionally contained 2 mM L-glutamine. The cells were maintained at 37 °C under 5% CO_2_ and 95% air.

### 2.3. MTT Assay

TCA8113 or HepG2 cells were seeded in 96-well plates at a density of 50,000 or 100,000 cells/well, respectively, 24 h prior to each experiment. The cells were exposed to melatonin or/and EGCG for 48 h. The medium was then removed and 200 μL RPMI-1640 medium containing 0.5 mg/mL MTT was added to each well. After a 4-h incubation, the medium was replaced with 150 μL dimethylsulfoxide. Optical density was measured by a microplate reader (Molecular Devices SpectraMax 190, Sunnyvale, CA, USA) at 490 nm. Data are presented as percentages of viable cells versus control.

### 2.4. Cell Migration

Cell migration was evaluated by scratch assay. TCA8113 cells or HepG2 cells were seeded on 6-well plates at a density of 1 × 10^6^/well. After 24 h incubation, sterile pipette tips were used to scratch cell layers. Following a brief wash with phosphate buffer solution (PBS), the cells were treated with melatonin or/and EGCG for 48 h. The cells were photographed, and the images were used to measure the width of the wound. Relative migration ratio of the scratched cells was calculated according to the formula: (width at 0 h − width at 48 h)/width at 0 h.

### 2.5. Colony Formation

TCA8113 or HepG2 cells were seeded in 6-cm cell culture dishes at a density of 500 or 40,000 cells/dish, respectively. After 24 h, the cells were treated with melatonin or/and EGCG. After a 14-day incubation, the cells were washed with PBS, fixed with the methanol for 15 min, and stained with 0.1% crystal violet for 20 min for counting colony number.

### 2.6. Western Blot

Cells cultured in 10-cm dishes were washed thrice with PBS and then lysed in Radio-Immune Precipitation Assay (RIPA) lysis buffer containing 1% PMSF on ice for 30 min. Following centrifugation (15,000× *g*, 10 min, 4 °C), the supernatants were incubated at 95 °C for 10 min in 5×SDS-PAGE (sodium dodecyl sulfate polyacrylamide gel electrophoresis) loading buffer. After protein separation through electrophoresis on a 15% SDS-PAGE gel, proteins in the gel were transferred to a Polyvinylidene difluoride (PVDF) membrane. The membrane was blocked using 5% nonfat dried milk in TBS-T (10 mM Tris-HCl, pH7.8, 150 mM NaCl and 0.05% Tween-20 (*v*/*v*)) for at least 2 h at room temperature. After a short wash with the TBS-T, the membrane was incubated with a primary antibody diluted in the TBS-T by 1000- to 5000-fold for at least 3 h at room temperature. The membrane was washed thrice with the TBS-T and then was incubated with a secondary antibody diluted in the TBS-T by 2500- to 5000-fold for 1 h at room temperature. Following three washings with the TBS-T, protein signals were detected using ChemiDoc XRS^+^ detection system (ECL, Bio-Rad, Hercules, CA, USA). The Quantity One^®^ Image Analyzer software program (Bio-Rad) was used for quantitative densitometric analysis.

### 2.7. Preparation of Nuclear Fraction

Cell nuclear fraction was prepared according to the protocol described in our previous study [51]. With minor modifications. In brief, cells were washed thrice with cold PBS containing 1 mM EDTA-Na_2_ and lysed for 20 min at 4 °C in cold isotonic buffer (pH 7.0) containing 250 mM sucrose, 20 mM HEPES, 0.15 mM EDTA, 0.015 mM EGTA, 1 mM dithiothreitol, 10 mM KCl, 1 mM phenylmethylsulfonyl fluoride, 20 mM NaF, 1 mM sodium pyrophosphate, 1 mM Na_3_VO_4_, 1% (*v*/*v*) Nonidet P-40 and 1 μg/mL protease inhibitor cocktail. The homogenates were centrifuged at 2000× *g* for 10 min at 4 °C. The supernatants were discarded and the precipitates were washed once with the above isotonic buffer but without containing the protease inhibitor cocktail. The nuclear pellets were sonicated in RIPA buffer containing 1% PMSF on ice for 5 min. The homogenates were centrifuged at 15,000× *g* for 15 min and the supernatants were collected as nuclear fraction. Proteins in the nuclear fraction were assessed using Western blot as described above. Validation of the purity of the cellular fractions is provided as Supplementary Figure S1.

### 2.8. Quinoprotein Measurement

Cell quinoprotein levels were measured essentially according to our previous protocol used for animal tissues [52]. Briefly, cells cultured in 10-cm dishes were washed thrice with PBS and then lysed in RIPA buffer containing 1% PMSF on ice for 30 min. After a centrifugation (15,000× *g*, 10 min, 4 °C), the supernatants were collected, and the protein concentrations were measured by BCA protein assay kit (Beyotime Biotechnology, Shanghai, China). The supernatants containing at least 0.5 mg protein were then mixed with 10% trichloroacetic acid at a volume ratio of 1/1 to precipitate soluble protein. After centrifuging at 1000× *g* for 10 min, the precipitate was washed with ethanol and then centrifuged at 4000× *g* for 5 min. The precipitate was further treated with chloroform-methanol (2:1, *v*/*v*) and centrifuged at 5000× *g* for 10 min. The resultant delipidated protein precipitate was suspended with 2 M potassium glycinate (pH 10.0) at 37 °C for 1 h and the supernatant was obtained by centrifuging at 15,000× *g* for 15 min. Finally, 4 μL NBT stock solution (12 mM) was added to 200 μL supernatant. The mixture was incubated at 37 °C in the dark, and absorbance at 530 nm was measured using the aforementioned microplate reader. Quinoprotein levels were expressed as OD_530 nm_/mg protein.

### 2.9. Analysis of mRNA Expression by Real-Time PCR

Total RNA was extracted by using TRIzol reagent (Takara Biotechnology, Kusatsu, Shiga, Japan) according to the manufacturer’s protocol. The preparation of the cDNA was carried out following the instructions described in the kit manual (RT-for-PCR kit, Takara Biotechnology). Real-Time Polymerase Chain Reaction (RT-PCR) was performed with a CFX System (Bio-Rad) according to the manufacturer’s instructions (Takara Biotechnology). The mRNA level was normalized with β-actin as an internal reference. The following primers were used for PCR reactions.

Human p53

Forward primer: 5′-3′-GGC CCA CTT CAC CGT ACT AA

Reverse primer: 5′-3′-GTG GTT TCA AGG CCA GAT GT

Human β-actin

Forward primer: 5′-3′-CTA CCT CAT GAA GAT CCT CAC CGA

Reverse primer: 5′-3′-TTC TCC TTA ATG TCA CGC ACG ATT

### 2.10. Statistical Analysis

Data are presented as means ± SEM. The significance differences between groups were examined by student’s t test, one-way ANOVA post hoc Dunnett test, or two-way ANOVA post hoc Bonferroni test, as appropriate. All statistical analyses were performed using GraphPad software (Prism version 5, San Diego, CA, USA). A *p* value of < 0.05 was considered statistically significant. We designed experiments and treatment groups based on the results of our previous experiments and did not do formal a priori power analysis.

## 3. Results

### 3.1. Melatonin-Induced p21 Correlates with the Increase of Nuclear Trx1 and TrxR1 in TCA8113 Cells

**Figure 1:**
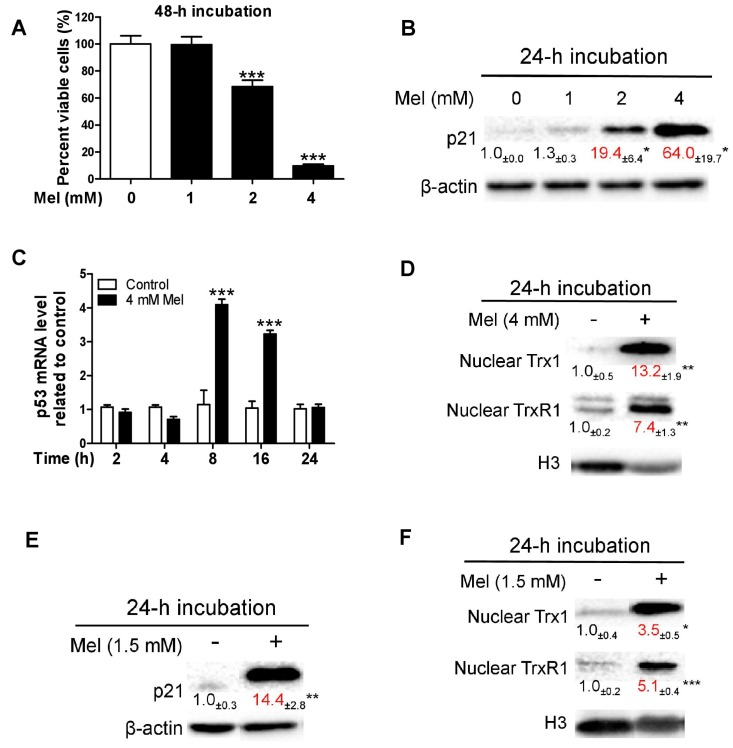
Influence of melatonin on cell viability, p21 and nuclear Trx1/TrxR1 in TCA8113 cells. (**A**) Cell viability. (**B**) The p21 protein. (**C**) Influence of 4 mM melatonin on p53 mRNA. (**D**) Influence of 4 mM melatonin on nuclear Trx1 and TrxR1. (**E**) Effect of 1.5 mM melatonin on p21 protein. (**F**) Effect of 1.5 mM melatonin on nuclear Trx1/TrxR1. Data are presented as mean ± SEM (*n* = 6 in A or 3 in B–F). Compared to the control, * *p* < 0.05, ** *p* < 0.01 and *** *p* < 0.001.

Confluent TCA8113 cells were treated with melatonin. Melatonin dose-dependently decreased viable cells at 48 h (Figure 1A) and increased p21 at 24 h (Figure 1B); this suggests that p21 is positively associated with melatonin-induced oncostatic activity. To elucidate how melatonin up-regulates p21, we detected the alteration of p53 mRNA. A dose of 4 mM melatonin was used because it substantially reduced the number of viable cells and dramatically increased p21 (Figure 1A,B). Melatonin transiently increased p53 mRNA within 24 h (Figure 1C). It is known that cisplatin stimulates translocation of Trx1 from the cytosol into the nucleus, leading to augmentation of p21 trans-activation via Trx1-dependent redox regulation of p53 [53,54]. We thus evaluated the influence of melatonin on nuclear Trx1 and its reductase (TrxR1). At 24 h, nuclear Trx1 and TrxR1 levels were dramatically increased by 4 mM melatonin (Figure 1D). To the best of our knowledge, this is the first finding that melatonin can stimulate nuclear translocation of Trx1 or TrxR1. Because this concept was established using high concentration of melatonin which initiates pronounced cytotoxic response, we subsequently investigated the influence of a lower concentration of melatonin (that caused marginal cytotoxicity) on p21 and nuclear Trx1/TrxR1. For this purpose, 1.5 mM melatonin was employed according to the dose response shown in Figure 1A. The low concentration of melatonin also significantly increased p21 (Figure 1E) and nuclear Trx1 and TrxR1 (Figure 1F) at 24 h. Cytoplasmic TrxR1 remained basically unchanged but cytoplasmic Trx1 significantly reduced following the melatonin treatment (Supplementary Figure S2). Overall, melatonin, at typical oncostatic concentrations, readily promotes nuclear translocation of both Trx1 and TrxR1, leading to avid up-regulation of p21 in TCA8113 cells.

### 3.2. EGCG Increases Quinoprotein Levels in TCA8113 Cells

**Figure 2:**
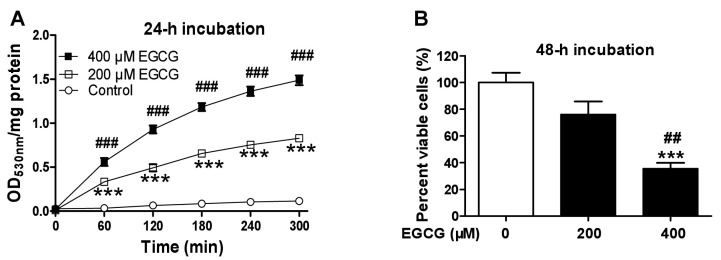
Influence of EGCG on quinoprotein and cell viability in TCA8113 cells. (**A**) Quinoprotein. (**B**) Cell viability. Data are presented as mean ± SEM (*n* = 3 in A or 6 in B). Compared to the control, *** *p* < 0.001. Compared to 200 μM EGCG, ## *p* < 0.01 and ### *p* < 0.001.

Auto-oxidation of EGCG produces ROS and EGCG quinone, which can covalently bind to the free thiol group of cysteine residues in proteins, resulting in the formation of quinoproteins and consequent alterations of protein functions [30,55]. Intracellular quinoprotein levels of cancer cells subjected to EGCG treatments have never been investigated. Thus, herein we evaluated the influence of EGCG on quinoprotein levels in TCA8113 cells. The derivative product of quinoproteins and NBT can be kinetically assessed at 530 nm [52]. With this approach, we found that EGCG dose-dependently increased quinoprotein levels following a 24-h incubation (Figure 2A). Based on the area under curve, EGCG at 400 μM raised quinoprotein levels 12.2-fold, whereas EGCG at 200 μM elevated quinoprotein levels 6.3-fold, relative to control. It has been demonstrated that the amounts of intracellular EGCG are no longer measured after 3 h in EGCG-treated cancer cells [56]. In this regard, quinoprotein containing the molecular skeleton of EGCG stands out as a biomarker signature because of the straightforward dose response relationship at apparently protracted time point. EGCG caused marginal cytotoxicity at 200 μM and reduced the number of viable cells by 65% at 400 μM (Figure 2B). Quinoprotein levels are apparently associated with EGCG-triggered oncostatic effect.

### 3.3. Concurrent Increases of p21 and Quinoprotein in TCA8113 Cells Treated with Melatonin and EGCG

**Figure 3:**
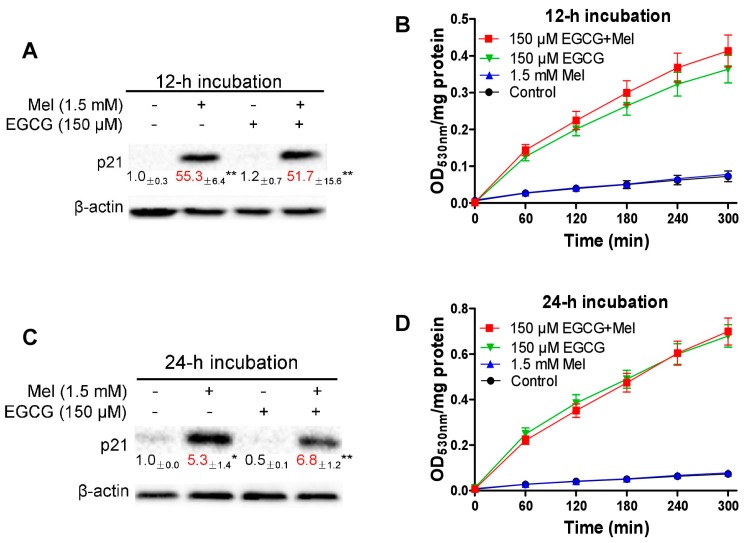

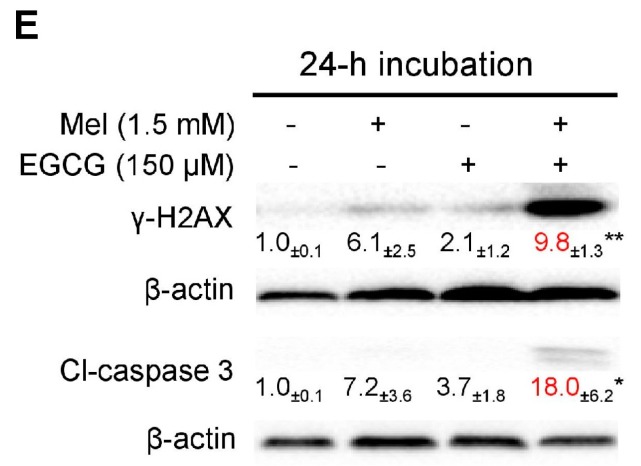
Influence of melatonin and EGCG and the combination on p21, quinoprotein, γ-H2AX and cleaved caspase 3 in TCA8113 cells. (**A**) 12-h p21. (**B**) 12-h quinoprotein. (**C**) 24-h p21. (**D**) 24-h quinoprotein. (**E**) 24-h γ-H2AX and cleaved caspase 3 (Cl-caspase 3). Data are presented as mean ± SEM (*n* = 3). Compared to the control, * *p* < 0.05, ** *p* < 0.01.

**Figure 4:**
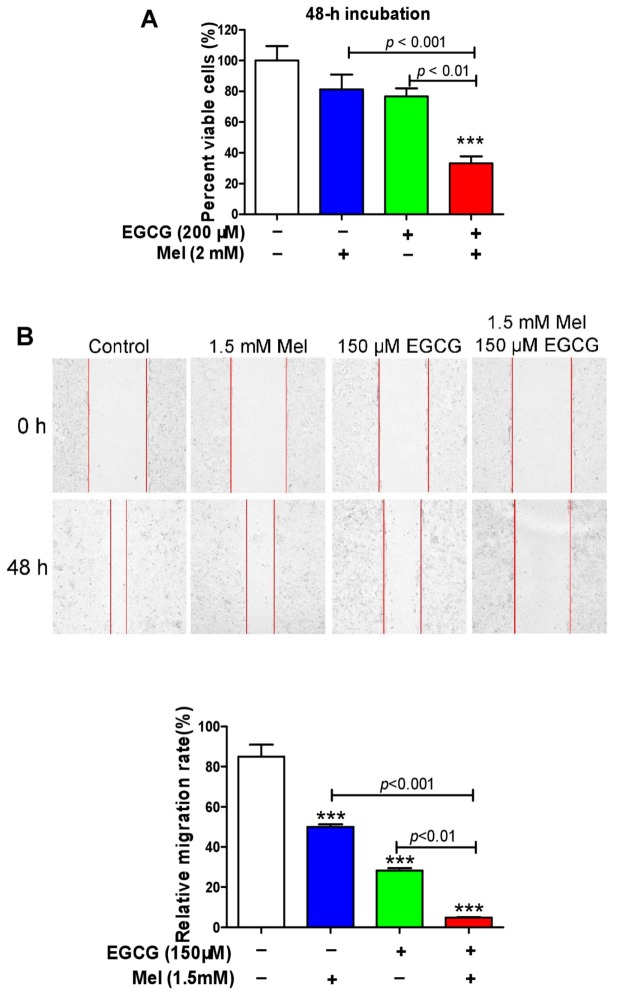
Influence of melatonin, EGCG and the combination on cell viability and migration in TCA8113 cells. (**A**) Cell viability measured by MTT assays. (**B**) Cell migration assessed by scratch assay. Data are presented as mean ± SEM (*n* = 6 in A or 3 in B). Compared to the control, *** *p* < 0.001.

**Figure 5:**
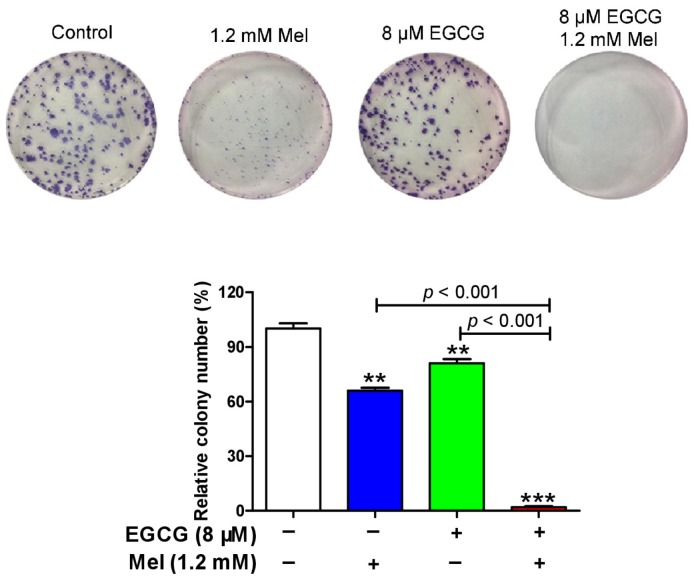
Influence of melatonin, EGCG and the combination on colony formation in TCA8113 cells. Drug was incubated with the cells for 14 day. Data are presented as mean ± SEM (*n* = 3). Compared to control, ** *p* < 0.01 and *** *p* < 0.001.

We examined the combined effects of melatonin and EGCG on p21 and quinoprotein. We found that EGCG did not influence the p21 protein up-regulation induced by melatonin and melatonin did not affect the dramatic increase of quinoproteins caused by EGCG at either 12 h (Figure 3A,B) or 24 h (Figure 3C,D). Because the above experiments showed that p21 and quinoprotein levels were positively associated with melatonin- and EGCG-induced oncostatic effects, respectively, the concurrent increase of p21 and quinoprotein by the co-treatment suggests that the two agents could generate enhanced oncostatic actions. This concept was reinforced by the following experiments using TCA8113 cells. The co-treatment was more efficient than the single agent in (1) increasing DNA damage as reflected by γ-H2AX (Figure 3E), (2) inducing apoptotic response as suggested by cleaved caspase 3 (Figure 3E), (3) decreasing the number of viable cells assessed by MTT assay (Figure 4A), (4) reducing cell migration evaluated by the wound healing assay (Figure 4B), and (5) inhibiting colony formation (Figure 5).

### 3.4. Melatonin Reduces p21 in HepG2 Cells and Sensitizes the Cells to EGCG

**Figure 6:**
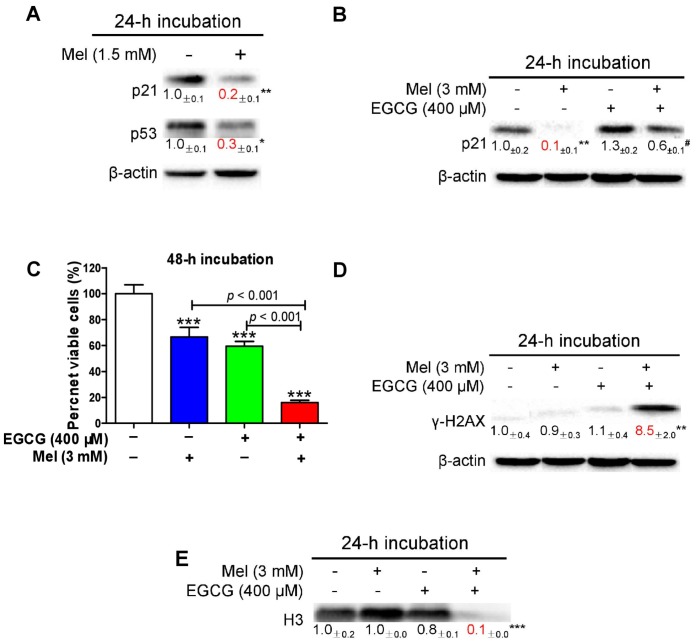
Influence of melatonin, EGCG and the combination on cell viability, p21, γ-H2AX and histone H3 in HepG2 cells. (**A**) Influence of 1.5 mM melatonin on p21 and p53. (**B**) Influence of 3 mM melatonin, 400 μM EGCG and the combination on p21. (**C**) Influence of 3 mM melatonin, 400 μM EGCG and the combination on cell viability measured by MTT assay. (**D**) Influence of 3 mM melatonin, 400 μM EGCG and the combination on γ-H2AX. (**E**) Influence of 3 mM melatonin, 400 μM EGCG and the combination on histone H3 (H3). Data are presented as mean ± SEM (*n* = 6 in C or 3 in A, B, D, E). Compared to the control, * *p* < 0.05, ** *p* < 0.01 and *** *p* < 0.001. Compared to 400 μM EGCG, # *p* < 0.05.

**Figure 7:**
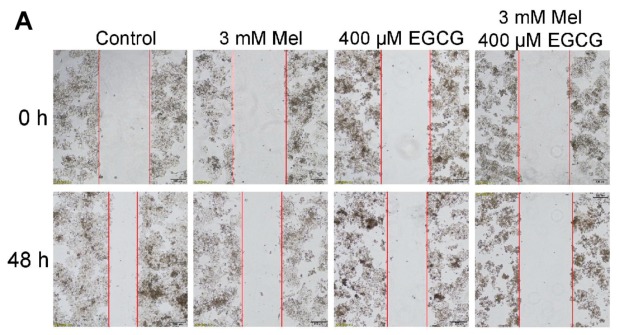

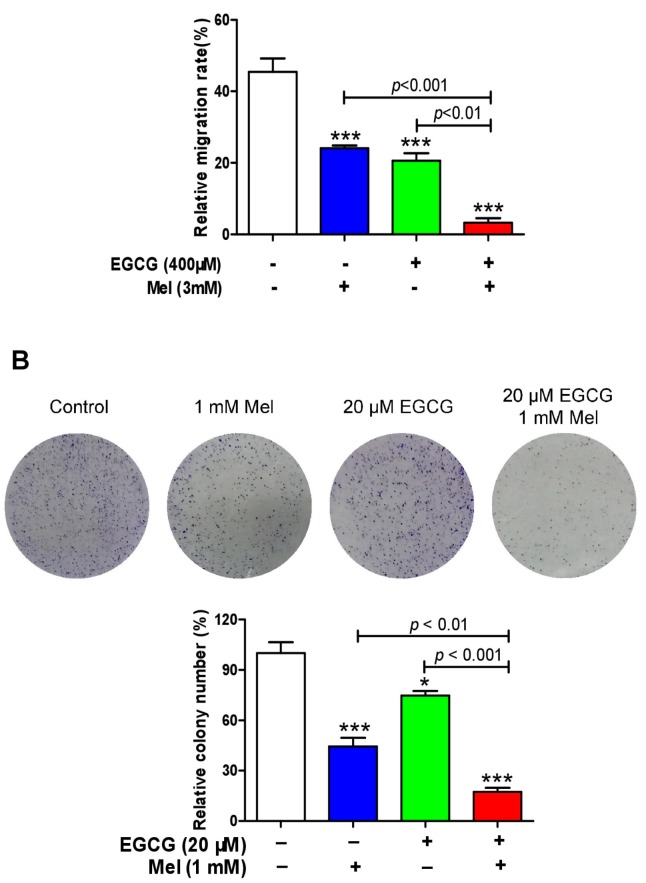
Influence of melatonin, EGCG and the combination on cell migration and colony formation in HepG2 cells. (**A**) Cell migration assessed by scratch assay. (**B**) Colony formation (drug was incubated with the cells for 14 day). Data are presented as mean ± SEM (*n* = 3). Compared to the control, * *p* < 0.05 and *** *p* < 0.001.

We have demonstrated previously that melatonin reduces EGCG-triggered hepatotoxicity in mice [50]. Thus, we determined the influence of co-treatment of melatonin and EGCG on human HCC HepG2 cells. When HepG2 cells approached confluence, the cells were exposed to 1.5 mM melatonin for 24 h. The melatonin significantly suppressed p21 probably owing to melatonin-induced low expression of p53 (Figure 6A). In a 24-h incubation, 3 mM melatonin also substantially suppressed p21, whereas 400 μM EGCG appeared to increase p21; however, the melatonin significantly attenuated p21 elevation induced by the EGCG (Figure 6B). Many studies have shown that attenuation of p21 in certain types of cancer cells could sensitize them to chemotherapeutic agents [57,58,59,60,61]. In glioblastoma U-87 MG cells, taxol also appeared to induce p21, similar to the action of EGCG in HepG2 cells. A p21 antisense oligonucleotide, which could dramatically decrease the steady state levels of p21, reduced p21 induction by taxol and significantly enhanced taxol-induced apoptosis [62]. Thus, we inferred that melatonin may enhance the oncostatic effects of EGCG in HepG2 cells. Five lines of evidence demonstrated that the combination of melatonin and EGCG could achieve an enhanced oncostatic effects compared to each agent alone. (1) Either 400 μM EGCG or 3 mM melatonin significantly reduced the number of viable cells measured by the MTT assay, but the co-treatment was more effective than the single agent (Figure 6C). (2) Neither 400 μM EGCG nor 3 mM melatonin significantly caused DNA damage as indicated by γ-H2AX; only the co-treatment effectively damaged DNA (Figure 6D). (3) Neither 400 μM EGCG nor 3 mM melatonin significantly affected nuclear H3 levels; only the co-treatment dramatically reduced nuclear H3 levels (Figure 6E), suggesting that the nucleus was severely damaged by the co-treatment. (4) Either 400 μM EGCG or 3 mM melatonin significantly reduce cell migration evaluated by wound healing assay, but the co-treatment was more potent than each single agent (Figure 7A). (5) Either 20 μM EGCG or 1 mM melatonin significantly inhibited colony formation, but the co-treatment was more robust than each single agent (Figure 7B).

### 3.5. EGCG Reverses Melatonin-Induced Pro-Survival Responses and Melatonin Does Not Impair Death-Promoting Action of EGCG in HepG2 Cells

**Figure 8:**
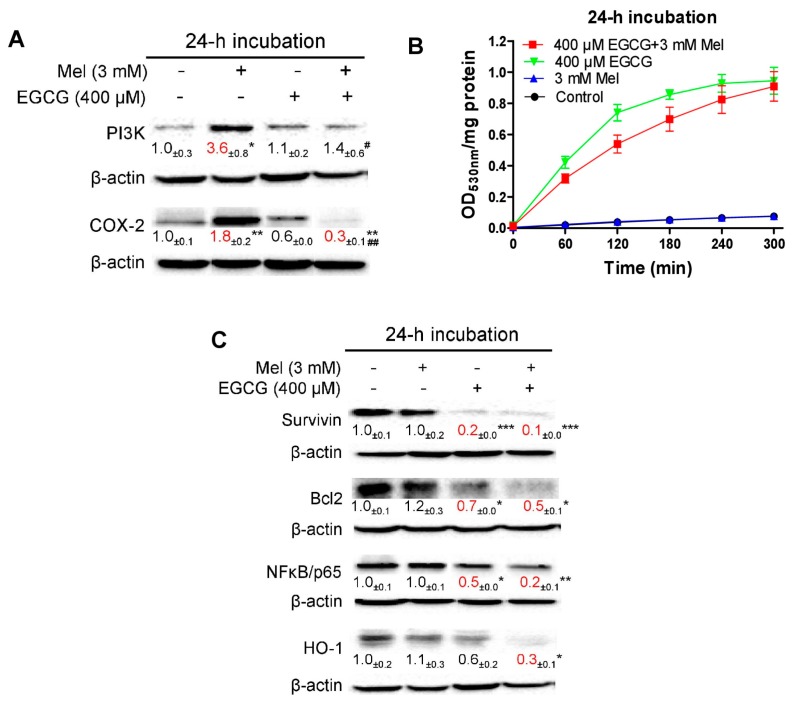
Influence of melatonin, EGCG and the combination on quinoprotein and proteins mainly associated with survival and apoptosis. (**A**) pro-survival PI3K and COX-2 proteins. (**B**) Quinoprotein. (**C**) Proteins associated survival with apoptosis. Data are presented as mean ± SEM (*n* = 3). Compared to control, * *p* < 0.05, ** *p* < 0.01 and *** *p* < 0.001. Compared to 3 mM melatonin, # *p* < 0.05 and ## *p* < 0.01.

In the aforementioned five experiments, except colony formation, the other studies were carried out using 3 mM melatonin and 400 μM EGCG. We further used these doses to examine the reciprocal interaction of melatonin and EGCG in HepG2 cells following a 24-h incubation. Melatonin treatment up-regulated pro-survival PI3K and COX-2 proteins (Figure 8A) probably as an adaptive response to pronounced reduction of p21 and moderate cytotoxixity (Figure 6B,C); these adaptive responses were thoroughly suppressed in the co-treatment of melatonin and EGCG (Figure 8A). As expected, the EGCG treatment dramatically increased quinoprotein levels, and the melatonin treatment did not significantly affect EGCG-elevated quinoprotein levels (Figure 8B). The EGCG treatment suppressed apoptosis inhibitor survivin, anti-apoptotic Bcl2, pro-survival transcriptional factor NFκB (Figure 8C). However, the co-treatment with melatonin enhanced the effects of EGCG (Figure 8C). Moreover, while the EGCG treatment seemed to suppress cytoprotective enzyme HO-1 (heme oxygenase-1), melatonin aided to significantly decrease HO-1 (Figure 8C).

## 4. Discussion

The present study using the assays of cytotoxicity, cell migration and colony formation reveals that the oncostatic activity of the co-treatment of melatonin and EGCG is always more robust than each single agent in two cancer cell lines with divergent p21 response to melatonin.

Early studies indicated that melatonin at a concentration as low as 1 nM significantly increased p21; thus, melatonin-induced increase of p21 is considered to be an important cancer-inhibitory mechanism [63,64]. The present study showed that melatonin at typical oncostatic doses markedly increased p21 in TCA8113 cells. The p21 up-regulation was correlated with Trx1/TrxR1 shuttling into the nucleus. Trx, TrxR and NADPH comprise a Trx system responsible for thiol-disulfide exchange reactions [65]. TrxR and Trx work together to reduce oxidized cysteine residues within the DNA-binding domain of several transcription factors including p53, maintaining maximal DNA-binding activity [66]. Transfection of Trx enhances p53-dependent expression of p21 [53]. Cisplatin-triggered nuclear translocation of Trx1 stimulates p53-dependent p21 trans-activation [53,54]. In estradiol-17β-treated cells, an alternative splicing variant of TrxR1 was found in the nucleus [67]. However, none of these studies revealed a concurrent nuclear translocation of Trx1 and TrxR1. We found that melatonin at oncostatic concentrations potently increased both nuclear Trx1 and nuclear TrxR1 levels in TCA8113 cells.

EGCG readily undergoes auto-oxidation to produce ROS and EGCG quinone, resulting in oxidative stress and formation of quinoproteins [30,55]. Such a prooxidant action of EGCG is viewed as a crucial anti-cancer mechanism [27,28,55,68]. Although the cancer-inhibitory effect of EGCG has been extensively studied [27,28,68], never has quinoprotein been examined in cancer cells subjected to EGCG treatment. The covalent binding of highly reactive EGCG quinone and protein containing free cysteine residue(s) leads to stable accumulation of quinoproteins, suggesting that quinoprotein is a biomarker signature of EGCG and could mirror EGCG-produced oxidative stress. The current study showed that p21 and quinoprotein levels were positively associated with melatonin- and EGCG-induced cell death, respectively. Cancer cell death caused by melatonin or EGCG is believed to be a consequence of oxidative stress [4,69,70]. Thus, p21 and quinoprotein could respectively reflect melatonin- and EGCG-triggered oxidative stress in TCA8113 cells. The concomitant increase of p21 and quinoprotein levels after the co-treatment indicated that two distinct anti-cancer routes resulted from each single agent were integrated and suggest that the two agents together could generate enhanced anti-cancer activity.

Although the up-regulation of p21 by melatonin is a putative anti-cancer mechanism, the down-regulation of p21 by melatonin at oncostatic concentrations has been shown in rat glioma cells and human gastric cancer cells [71,72]. However, the biological importance of such an opposite effect of melatonin on p21 is poorly understood. It is well known that p21 has a strong inhibitory effect on cell cycle; however, it also plays a powerful anti-apoptotic role in some cancer cells and confers these cells with a survival advantage, making it an appropriate target for suppression by pharmacological agents [57,73]. Inhibition of p21 by antisense oligodeoxynucleotide causes apoptosis in human breast cancer cells [74,75]. Compelling evidence demonstrates that attenuation of p21 in cancer cells make a battery of DNA-damaging chemotherapeutic agents more effective [57,58,59,60,61]. HCC is a highly malignant tumor and is refractory to most chemotherapeutic agents [76]. Sorafenib is a first-line chemotherapeutic drug for patients with advanced HCC [77]. It has been documented that sorafenib can markedly reduce p21 levels in tumors or cancer cells including HepG2 cells [78,79], suggesting that p21 suppression plays a role in for HCC therapy. The present study showed that melatonin dramatically suppressed p21 in HepG2 cells, which could be ascribed to melatonin-induced reduction of p53 (Figure 6A). We further found that melatonin-induced reduction of p53 in HepG2 cells was associated with melatonin-triggered increase of MDM2 (Supplementary Figure S3). Thus, melatonin-induced reduction of p53 possibly involved p53 degradation by proteasome via MDM2-mediated ubiquitination of p53. Currently there is ample evidence indicating that melatonin is a highly appealing candidate for modulating HCC to achieve a successful chemotherapy [80,81,82,83,84,85]. In this setting, the down-regulation of p21 by melatonin as evidenced by the present study may play a pivotal role. Thus, the pursuit using melatonin as an adjunct agent for HCC therapy may be promising. Two earlier studies indicated that melatonin up-regulated p21 in HepG2 cells [86,87], whereas the present study found the opposite effect. The discrepancy may result from different cell density and incubation time. We carried out the experiments when HepG2 reached confluence and investigated the influence of a 24-h incubation of melatonin on p21. The results that melatonin increased p21 [86,87] were obtained at much higher cell density after a 48-h incubation.

The present study showed that while melatonin down-regulated p21 and caused moderate cytotoxicity, pro-survival PI3K and COX-2 proteins were up-regulated in HepG2 cells. Perhaps such a drug resistant response attenuated the cytotoxic effect of melatonin. EGCG reduced the expression of survivin, Bcl2 and NFκB. Nonetheless, oncostatic activity of EGCG by itself was inadequate. When the drugs were combined, (1) melatonin probably sensitized HepG2 cells to EGCG toxicity via suppressing p21; (2) EGCG inhibited melatonin-induced pro-survival responses; (3) COX-2 and HO-1 were significantly suppressed only by the co-treatment; and (4) melatonin aided EGCG to achieve an increased inhibition of Bcl2 (from 30% to 50%) and NFκB (from 50% to 80%). These molecular pathways together resulted in enhanced cytotoxicity in the co-treatment.

## 5. Conclusions

EGCG has shown anti-cancer effects in many studies. However, high-dose EGCG is known to cause hepatotoxicity. Our previous study has demonstrated that melatonin reduces hepatotoxicity triggered by high-dose EGCG [50], making intake of EGCG at high levels more tolerable. The present study reveals that co-treatment of melatonin and EGCG in cancer cells is more potent than each single agent in reducing the number of viable cells, inhibiting cell migration and suppressing colony formation. Taking both the safety and anti-cancer effect of EGCG into account, melatonin plays dual roles in reducing hepatotoxicity risk of EGCG and increasing the anti-cancer potential of EGCG; thus, melatonin and EGCG appear to be ideal partners in fighting cancer.

## Supplementary Materials

The following are available online at https://www.mdpi.com/2073-4409/8/7/745/s1, Figure S1: Validation of the purity of the cellular fractions by using TCA8113 cells (*n* = 6). Figure S2: Cytoplasmic TrxR1 and Trx1 in response to melatonin treatment in TCA8113 cells. Data are presented as mean ± SEM (*n* = 6). Compared to the control, * *p* < 0.05. Figure S3: The expression of MDM2 and p53 proteins in HepG2 cells treated with melatonin. Data are presented as mean ± SEM (*n* = 3). Compared to the control, ** *p* < 0.01 and *** *p* < 0.001.

## References

[1] Maitra S., Bhattacharya D., Das S., Bhattacharya S. (2019). Melatonin and its anti-glioma functions: A comprehensive review. Rev. Neurosci..

[2] Reiter R.J., Mayo J.C., Tan D.X., Sainz R.M., Alatorre-Jimenez M., Qin L. (2016). Melatonin as an antioxidant: Under promises but over delivers. J. Pineal Res..

[3] Manchester L.C., Coto-Montes A., Boga J.A., Andersen L.P., Zhou Z., Galano A., Vriend J., Tan D.X., Reiter R.J. (2015). Melatonin: An ancient molecule that makes oxygen metabolically tolerable. J. Pineal Res..

[4] Zhang H.M., Zhang Y. (2014). Melatonin: A well-documented antioxidant with conditional pro-oxidant actions. J. Pineal Res..

[5] Reiter R.J., Rosales-Corral S.A., Tan D.X., Acuna-Castroviejo D., Qin L., Yang S.F., Xu K. (2017). Melatonin, a Full Service Anti-Cancer Agent: Inhibition of Initiation, Progression and Metastasis. Int. J. Mol. Sci..

[6] Reiter R.J. (2004). Mechanisms of cancer inhibition by melatonin. J. Pineal Res..

[7] Tan D.X., Manchester L.C., Terron M.P., Flores L.J., Reiter R.J. (2007). One molecule, many derivatives: A never-ending interaction of melatonin with reactive oxygen and nitrogen species?. J. Pineal Res..

[8] Rodriguez C., Mayo J.C., Sainz R.M., Antolin I., Herrera F., Martin V., Reiter R.J. (2004). Regulation of antioxidant enzymes: A significant role for melatonin. J. Pineal Res..

[9] Vriend J., Reiter R.J. (2015). The Keap1-Nrf2-antioxidant response element pathway: A review of its regulation by melatonin and the proteasome. Mol. Cell Endocrinol..

[10] Trachootham D., Zhou Y., Zhang H., Demizu Y., Chen Z., Pelicano H., Chiao P.J., Achanta G., Arlinghaus R.B., Liu J. (2006). Selective killing of oncogenically transformed cells through a ROS-mediated mechanism by beta-phenylethyl isothiocyanate. Cancer Cell.

[11] Jaramillo M.C., Zhang D.D. (2013). The emerging role of the Nrf2-Keap1 signaling pathway in cancer. Genes Dev..

[12] Raninga P.V., Trapani G.D., Tonissen K.F. (2014). Cross Talk between Two Antioxidant Systems, Thioredoxin and DJ-1: Consequences for Cancer. Oncoscience.

[13] Holmgren A. (2006). Selenite in cancer therapy: A commentary on “Selenite induces apoptosis in sarcomatoid malignant mesothelioma cells through oxidative stress”. Free Radic. Biol. Med..

[14] Sun K., Wu S., Wang Y., Wan X., Thompson H.J., Zhang J. (2013). High-dose sodium selenite toxicity cannot be prevented by the co-administration of pharmacological levels of epigallocatechin-3-gallate which in turn aggravates the toxicity. Food Chem. Toxicol..

[15] Andersen L.P., Gogenur I., Rosenberg J., Reiter R.J. (2016). The Safety of Melatonin in Humans. Clin. Drug Investig..

[16] Reiter R.J., Tan D.X., Sainz R.M., Mayo J.C., Lopez-Burillo S. (2002). Melatonin: Reducing the toxicity and increasing the efficacy of drugs. J. Pharm. Pharmacol..

[17] Majidinia M., Sadeghpour A., Mehrzadi S., Reiter R.J., Khatami N., Yousefi B. (2017). Melatonin: A pleiotropic molecule that modulates DNA damage response and repair pathways. J. Pineal Res..

[18] Li Y., Li S., Zhou Y., Meng X., Zhang J.J., Xu D.P., Li H.B. (2017). Melatonin for the prevention and treatment of cancer. Oncotarget.

[19] Galley H.F., McCormick B., Wilson K.L., Lowes D.A., Colvin L., Torsney C. (2017). Melatonin limits paclitaxel-induced mitochondrial dysfunction in vitro and protects against paclitaxel-induced neuropathic pain in the rat. J. Pineal Res..

[20] Pariente R., Pariente J.A., Rodriguez A.B., Espino J. (2016). Melatonin sensitizes human cervical cancer HeLa cells to cisplatin-induced cytotoxicity and apoptosis: Effects on oxidative stress and DNA fragmentation. J. Pineal Res..

[21] Manda K., Ueno M., Anzai K. (2009). Cranial irradiation-induced inhibition of neurogenesis in hippocampal dentate gyrus of adult mice: Attenuation by melatonin pretreatment. J. Pineal Res..

[22] Ortiz F., Acuna-Castroviejo D., Doerrier C., Dayoub J.C., Lopez L.C., Venegas C., Garcia J.A., Lopez A., Volt H., Luna-Sanchez M. (2015). Melatonin blunts the mitochondrial/NLRP3 connection and protects against radiation-induced oral mucositis. J. Pineal Res..

[23] Alonso-Gonzalez C., Gonzalez A., Martinez-Campa C., Gomez-Arozamena J., Cos S. (2015). Melatonin sensitizes human breast cancer cells to ionizing radiation by downregulating proteins involved in double-strand DNA break repair. J. Pineal Res..

[24] Alonso-Gonzalez C., Gonzalez A., Martinez-Campa C., Menendez-Menendez J., Gomez-Arozamena J., Garcia-Vidal A., Cos S. (2016). Melatonin enhancement of the radiosensitivity of human breast cancer cells is associated with the modulation of proteins involved in estrogen biosynthesis. Cancer Lett..

[25] Casado-Zapico S., Rodriguez-Blanco J., Garcia-Santos G., Martin V., Sanchez-Sanchez A.M., Antolin I., Rodriguez C. (2010). Synergistic antitumor effect of melatonin with several chemotherapeutic drugs on human Ewing sarcoma cancer cells: Potentiation of the extrinsic apoptotic pathway. J. Pineal Res..

[26] Han M., Zhao G., Wang Y., Wang D., Sun F., Ning J., Wan X., Zhang J. (2016). Safety and anti-hyperglycemic efficacy of various tea types in mice. Sci. Rep..

[27] Yang C.S., Hong J. (2013). Prevention of chronic diseases by tea: Possible mechanisms and human relevance. Annu. Rev. Nutr..

[28] Yang C.S., Wang X., Lu G., Picinich S.C. (2009). Cancer prevention by tea: Animal studies, molecular mechanisms and human relevance. Nat. Rev. Cancer.

[29] Dong R., Wang D., Wang X., Zhang K., Chen P., Yang C.S., Zhang J. (2016). Epigallocatechin-3-gallate enhances key enzymatic activities of hepatic thioredoxin and glutathione systems in selenium-optimal mice but activates hepatic Nrf2 responses in selenium-deficient mice. Redox Biol..

[30] Wei Y., Chen P., Ling T., Wang Y., Dong R., Zhang C., Zhang L., Han M., Wang D., Wan X. (2016). Certain (−)-epigallocatechin-3-gallate (EGCG) auto-oxidation products (EAOPs) retain the cytotoxic activities of EGCG. Food Chem..

[31] Wang D., Taylor E.W., Wang Y., Wan X., Zhang J. (2012). Encapsulated nanoepigallocatechin-3-gallate and elemental selenium nanoparticles as paradigms for nanochemoprevention. Int. J. Nanomed..

[32] Pae M., Ren Z., Meydani M., Shang F., Smith D., Meydani S.N., Wu D. (2012). Dietary supplementation with high dose of epigallocatechin-3-gallate promotes inflammatory response in mice. J. Nutr. Biochem..

[33] Shanafelt T.D., Call T.G., Zent C.S., LaPlant B., Bowen D.A., Roos M., Secreto C.R., Ghosh A.K., Kabat B.F., Lee M.J. (2009). Phase I trial of daily oral Polyphenon E in patients with asymptomatic Rai stage 0 to II chronic lymphocytic leukemia. J. Clin. Oncol..

[34] Bitzer Z.T., Elias R.J., Vijay-Kumar M., Lambert J.D. (2016). (−)-epigallocatechin-3-gallate decreases colonic inflammation and permeability in a mouse model of colitis, but reduces macronutrient digestion and exacerbates weight loss. Mol. Nutr. Food Res..

[35] Lambert J.D., Kennett M.J., Sang S., Reuhl K.R., Ju J., Yang C.S. (2010). Hepatotoxicity of high oral dose (−)-epigallocatechin-3-gallate in mice. Food Chem. Toxicol..

[36] James K.D., Forester S.C., Lambert J.D. (2015). Dietary pretreatment with green tea polyphenol, (−)-epigallocatechin-3-gallate reduces the bioavailability and hepatotoxicity of subsequent oral bolus doses of (−)-epigallocatechin-3-gallate. Food Chem. Toxicol..

[37] Mazzanti G., Menniti-Ippolito F., Moro P.A., Cassetti F., Raschetti R., Santuccio C., Mastrangelo S. (2009). Hepatotoxicity from green tea: A review of the literature and two unpublished cases. Eur. J. Clin. Pharmacol..

[38] Mazzanti G., Di Sotto A., Vitalone A. (2015). Hepatotoxicity of green tea: An update. Arch. Toxicol..

[39] Yates A.A., Erdman J.W., Shao A., Dolan L.C., Griffiths J.C. (2017). Bioactive nutrients—Time for tolerable upper intake levels to address safety. Regul. Toxicol. Pharmacol..

[40] Dekant W., Fujii K., Shibata E., Morita O., Shimotoyodome A. (2017). Safety assessment of green tea based beverages and dried green tea extracts as nutritional supplements. Toxicol. Lett..

[41] Hu J., Webster D., Cao J., Shao A. (2018). The safety of green tea and green tea extract consumption in adults—Results of a systematic review. Regul. Toxicol. Pharmacol..

[42] Setiawan V.W., Zhang Z.F., Yu G.P., Lu Q.Y., Li Y.L., Lu M.L., Wang M.R., Guo C.H., Yu S.Z., Kurtz R.C. (2001). Protective effect of green tea on the risks of chronic gastritis and stomach cancer. Int. J. Cancer.

[43] Mu L.N., Lu Q.Y., Yu S.Z., Jiang Q.W., Cao W., You N.C., Setiawan V.W., Zhou X.F., Ding B.G., Wang R.H. (2005). Green tea drinking and multigenetic index on the risk of stomach cancer in a Chinese population. Int. J. Cancer.

[44] Sasazuki S., Inoue M., Hanaoka T., Yamamoto S., Sobue T., Tsugane S. (2004). Green tea consumption and subsequent risk of gastric cancer by subsite: The JPHC Study. Cancer Causes Control.

[45] Jian L., Xie L.P., Lee A.H., Binns C.W. (2004). Protective effect of green tea against prostate cancer: A case-control study in southeast China. Int. J. Cancer.

[46] Kurahashi N., Sasazuki S., Iwasaki M., Inoue M., Tsugane S., Group J.S. (2008). Green tea consumption and prostate cancer risk in Japanese men: A prospective study. Am. J. Epidemiol..

[47] Yang G., Shu X.O., Li H., Chow W.H., Ji B.T., Zhang X., Gao Y.T., Zheng W. (2007). Prospective cohort study of green tea consumption and colorectal cancer risk in women. Cancer Epidemiol. Biomark. Prev..

[48] Zhang M., Holman C.D., Huang J.P., Xie X. (2007). Green tea and the prevention of breast cancer: A case-control study in Southeast China. Carcinogenesis.

[49] Zhong L., Goldberg M.S., Gao Y.T., Hanley J.A., Parent M.E., Jin F. (2001). A population-based case-control study of lung cancer and green tea consumption among women living in Shanghai, China. Epidemiology.

[50] Wang D., Wei Y., Wang T., Wan X., Yang C.S., Reiter R.J., Zhang J. (2015). Melatonin attenuates (−)-epigallocatehin-3-gallate-triggered hepatotoxicity without compromising its downregulation of hepatic gluconeogenic and lipogenic genes in mice. J. Pineal Res..

[51] Wang D., Wang Y., Wan X., Yang C.S., Zhang J. (2015). Green tea polyphenol (−)-epigallocatechin-3-gallate triggered hepatotoxicity in mice: Responses of major antioxidant enzymes and the Nrf2 rescue pathway. Toxicol. Appl. Pharmacol..

[52] Zhang K., Dong R., Sun K., Wang X., Wang J., Yang C.S., Zhang J. (2017). Synergistic toxicity of epigallocatechin-3-gallate and diethyldithiocarbamate, a lethal encounter involving redox-active copper. Free Radic. Biol. Med..

[53] Ueno M., Masutani H., Arai R.J., Yamauchi A., Hirota K., Sakai T., Inamoto T., Yamaoka Y., Yodoi J., Nikaido T. (1999). Thioredoxin-dependent redox regulation of p53-mediated p21 activation. J. Biol. Chem..

[54] Ueno M., Matsutani Y., Nakamura H., Masutani H., Yagi M., Yamashiro H., Kato H., Inamoto T., Yamauchi A., Takahashi R. (2000). Possible association of thioredoxin and p53 in breast cancer. Immunol. Lett..

[55] Ishii T., Mori T., Tanaka T., Mizuno D., Yamaji R., Kumazawa S., Nakayama T., Akagawa M. (2008). Covalent modification of proteins by green tea polyphenol (−)-epigallocatechin-3-gallate through autoxidation. Free Radic. Biol. Med..

[56] Tao L., Park J.Y., Lambert J.D. (2015). Differential prooxidative effects of the green tea polyphenol, (−)-epigallocatechin-3-gallate, in normal and oral cancer cells are related to differences in sirtuin 3 signaling. Mol. Nutr. Food Res..

[57] Weiss R.H. (2003). p21Waf1/Cip1 as a therapeutic target in breast and other cancers. Cancer Cell.

[58] Fan S., Chang J.K., Smith M.L., Duba D., Fornace A.J., O’Connor P.M. (1997). Cells lacking CIP1/WAF1 genes exhibit preferential sensitivity to cisplatin and nitrogen mustard. Oncogene.

[59] Lazzarini R., Moretti S., Orecchia S., Betta P.G., Procopio A., Catalano A. (2008). Enhanced antitumor therapy by inhibition of p21waf1 in human malignant mesothelioma. Clin. Cancer Res..

[60] Stewart Z.A., Mays D., Pietenpol J.A. (1999). Defective G1-S cell cycle checkpoint function sensitizes cells to microtubule inhibitor-induced apoptosis. Cancer Res..

[61] Waldman T., Lengauer C., Kinzler K.W., Vogelstein B. (1996). Uncoupling of S phase and mitosis induced by anticancer agents in cells lacking p21. Nature.

[62] Li Y., Dowbenko D., Lasky L.A. (2002). AKT/PKB phosphorylation of p21Cip/WAF1 enhances protein stability of p21Cip/WAF1 and promotes cell survival. J. Biol. Chem..

[63] Cos S., Mediavilla M.D., Fernandez R., Gonzalez-Lamuno D., Sanchez-Barcelo E.J. (2002). Does melatonin induce apoptosis in MCF-7 human breast cancer cells in vitro?. J. Pineal Res..

[64] Proietti S., Cucina A., Dobrowolny G., D’Anselmi F., Dinicola S., Masiello M.G., Pasqualato A., Palombo A., Morini V., Reiter R.J. (2014). Melatonin down-regulates MDM2 gene expression and enhances p53 acetylation in MCF-7 cells. J. Pineal Res..

[65] Arner E.S. (2009). Focus on mammalian thioredoxin reductases--important selenoproteins with versatile functions. Biochim. Biophys. Acta.

[66] Heilman J.M., Burke T.J., McClain C.J., Watson W.H. (2011). Transactivation of gene expression by NF-kappaB is dependent on thioredoxin reductase activity. Free Radic. Biol. Med..

[67] Damdimopoulos A.E., Miranda-Vizuete A., Treuter E., Gustafsson J.A., Spyrou G. (2004). An alternative splicing variant of the selenoprotein thioredoxin reductase is a modulator of estrogen signaling. J. Biol. Chem..

[68] Yang C.S., Wang H. (2011). Mechanistic issues concerning cancer prevention by tea catechins. Mol. Nutr. Food Res..

[69] Forester S.C., Lambert J.D. (2011). The role of antioxidant versus pro-oxidant effects of green tea polyphenols in cancer prevention. Mol. Nutr. Food Res..

[70] Lambert J.D., Elias R.J. (2010). The antioxidant and pro-oxidant activities of green tea polyphenols: A role in cancer prevention. Arch. Biochem. Biophys..

[71] Martin V., Herrera F., Garcia-Santos G., Antolin I., Rodriguez-Blanco J., Medina M., Rodriguez C. (2007). Involvement of protein kinase C in melatonin’s oncostatic effect in C6 glioma cells. J. Pineal Res..

[72] Song J., Ma S.J., Luo J.H., Zhang H., Wang R.X., Liu H., Li L., Zhang Z.G., Zhou R.X. (2018). Melatonin induces the apoptosis and inhibits the proliferation of human gastric cancer cells via blockade of the AKT/MDM2 pathway. Oncol. Rep..

[73] Asada M., Yamada T., Ichijo H., Delia D., Miyazono K., Fukumuro K., Mizutani S. (1999). Apoptosis inhibitory activity of cytoplasmic p21(Cip1/WAF1) in monocytic differentiation. EMBO J..

[74] Fan Y., Borowsky A.D., Weiss R.H. (2003). An antisense oligodeoxynucleotide to p21(Waf1/Cip1) causes apoptosis in human breast cancer cells. Mol. Cancer Ther..

[75] Weiss R.H., Marshall D., Howard L., Corbacho A.M., Cheung A.T., Sawai E.T. (2003). Suppression of breast cancer growth and angiogenesis by an antisense oligodeoxynucleotide to p21(Waf1/Cip1). Cancer Lett..

[76] Zhu A.X. (2006). Systemic therapy of advanced hepatocellular carcinoma: How hopeful should we be?. Oncologist.

[77] Niu L., Liu L., Yang S., Ren J., Lai P.B.S., Chen G.G. (2017). New insights into sorafenib resistance in hepatocellular carcinoma: Responsible mechanisms and promising strategies. Biochim. Biophys. Acta.

[78] Huynh H., Lee J.W., Chow P.K., Ngo V.C., Lew G.B., Lam I.W., Ong H.S., Chung A., Soo K.C. (2009). Sorafenib induces growth suppression in mouse models of gastrointestinal stromal tumor. Mol. Cancer Ther..

[79] Inoue H., Hwang S.H., Wecksler A.T., Hammock B.D., Weiss R.H. (2011). Sorafenib attenuates p21 in kidney cancer cells and augments cell death in combination with DNA-damaging chemotherapy. Cancer Biol. Ther..

[80] Fan L., Sun G., Ma T., Zhong F., Lei Y., Li X., Wei W. (2013). Melatonin reverses tunicamycin-induced endoplasmic reticulum stress in human hepatocellular carcinoma cells and improves cytotoxic response to doxorubicin by increasing CHOP and decreasing survivin. J. Pineal Res..

[81] Prieto-Dominguez N., Ordonez R., Fernandez A., Mendez-Blanco C., Baulies A., Garcia-Ruiz C., Fernandez-Checa J.C., Mauriz J.L., Gonzalez-Gallego J. (2016). Melatonin-induced increase in sensitivity of human hepatocellular carcinoma cells to sorafenib is associated with reactive oxygen species production and mitophagy. J. Pineal Res..

[82] Lin S., Hoffmann K., Gao C., Petrulionis M., Herr I., Schemmer P. (2017). Melatonin promotes sorafenib-induced apoptosis through synergistic activation of JNK/c-jun pathway in human hepatocellular carcinoma. J. Pineal Res..

[83] Zha L., Fan L., Sun G., Wang H., Ma T., Zhong F., Wei W. (2012). Melatonin sensitizes human hepatoma cells to endoplasmic reticulum stress-induced apoptosis. J. Pineal Res..

[84] Hao J., Li Z., Zhang C., Yu W., Tang Z., Li Y., Feng X., Gao Y., Liu Q., Huang W. (2017). Targeting NF-kappaB/AP-2beta signaling to enhance antitumor activity of cisplatin by melatonin in hepatocellular carcinoma cells. Am. J. Cancer Res..

[85] Mortezaee K. (2018). Human hepatocellular carcinoma: Protection by melatonin. J. Cell. Physiol..

[86] Martin-Renedo J., Mauriz J.L., Jorquera F., Ruiz-Andres O., Gonzalez P., Gonzalez-Gallego J. (2008). Melatonin induces cell cycle arrest and apoptosis in hepatocarcinoma HepG2 cell line. J. Pineal Res..

[87] Carbajo-Pescador S., Martin-Renedo J., Garcia-Palomo A., Tunon M.J., Mauriz J.L., Gonzalez-Gallego J. (2009). Changes in the expression of melatonin receptors induced by melatonin treatment in hepatocarcinoma HepG2 cells. J. Pineal Res..

