# Supplementary material for: Melatonin and (−)-Epigallocatechin-3-Gallate: Partners in Fighting Cancer
Source: Cells. 2019 Jul 19;8(7):745. doi: 10.3390/cells8070745 (PMC6678710; doi:10.3390/cells8070745)
Supplement: Supplementary file 1 [file cells-08-00745-s001.pdf]

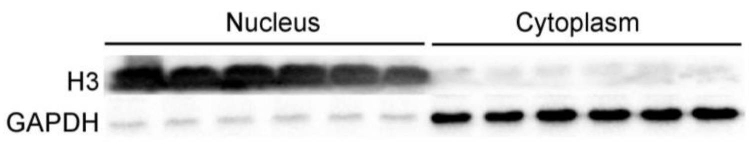

Supplementary Figure S1. Validation of the purity of the cellular fractions by using TCA8113 cells. (n = 6).

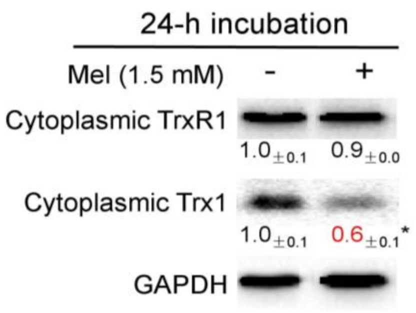

Supplementary Figure S2. Cytoplasmic TrxR1 and Trx1 in response to melatonin treatment in TCA8113 cells. Data are presented as mean ± SEM (n = 6). Compared to the control, * $p < 0.05$.

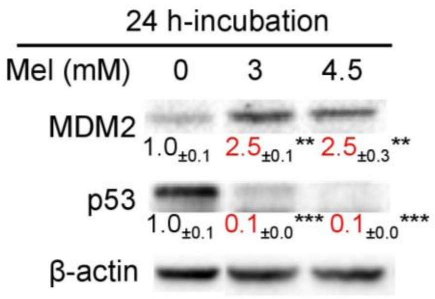

Supplementary Figure S3. The expression of MDM2 and p53 proteins in HepG2 cells treated with melatonin. Data are presented as mean ± SEM (n = 3). Compared to the control, ** $p < 0.01$ and *** $p < 0.001$.
